# Component Associations of the Healthy Worker Survivor Bias in Medical Radiation Workers

**DOI:** 10.1002/ajim.23727

**Published:** 2025-04-25

**Authors:** Won Jin Lee, Jaeho Jeong, Young Min Kim

**Affiliations:** ^1^ Department of Preventive Medicine Korea University College of Medicine Seoul South Korea; ^2^ Department of Statistics Kyungpook National University Daegu South Korea

**Keywords:** bias, cohort, health professionals, ionizing radiation, occupational exposure

## Abstract

**Background:**

The healthy worker survivor bias may vary by sex. This study investigated three component associations necessary for this bias to determine the origins of sex differences in this bias among male and female workers.

**Methods:**

We analyzed a data set of 93,918 South Korean diagnostic medical radiation workers registered in the National Dose Registry from 1996 to 2011, linked with mortality and cancer incidence data. Component associations were assessed using Cox regression to estimate hazard ratios (HRs) and logistic regression with generalized estimating equations to estimate odds ratios (ORs).

**Results:**

A significant association between prior cumulative exposure and employment status was observed for all‐cause mortality in male (HR 1.06, 95% CI 1.02–1.10), whereas an inverse association was noted in female workers (HR 0.82, 95% CI 0.78–0.87). Adjusted ORs for employment status and subsequent exposure for all‐cause mortality, as well as HRs for employment status and survival time, demonstrated associations in the same direction in both males and females.

**Conclusions:**

Our findings demonstrate that sex‐specific differences in healthy worker survivor bias were primarily driven by the association between prior exposure and employment status. To improve bias mitigation in occupational cohort studies, sex‐specific components should be incorporated.

## 1 Introduction

**Figure 1:**
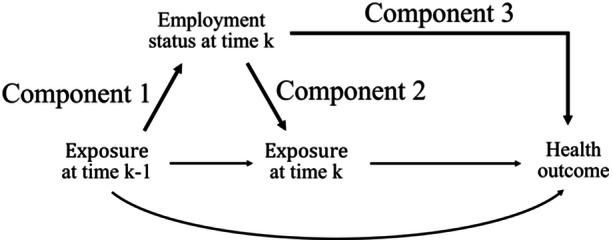
A simplified causal diagram representing the healthy worker survivor bias. Exposure status is defined as a 10‐year latency period cumulative dose > mSv at time *k* (yes/no). Employment status is a time‐varying confounder indicating whether a worker employed at time *k* (yes/no). Health outcome represents death or cancer incidence. C1, component 1; C2, component 2; C3, component 3.

The healthy worker survivor bias can be identified through three component associations using a causal diagram: the association between prior exposure and employment status (C1), the association between current employment status and subsequent exposure (C2), and the association between employment status and the outcome of interest (C3) (Figure 1) [1]. Several studies have investigated these structural components to assess evidence of healthy worker survivor bias, including those conducted on textile manufacturing workers [1], autoworkers [2], boat building industry workers [3], white‐collar workers [4], and workers exposed to silica dust [5] or acrylonitrile [6].

While various factors can modify the healthy worker survivor effect [7], little is known about how this effect differs by sex. Previous studies have reported a weaker healthy worker effect among women in the United States [8, 9] and Japan [10]. Conversely, stronger effects have been observed for causes of death among European female workers compared to male workers [11]. Recently, we reported an opposite pattern of the healthy worker survivor effect between men and women for occupational radiation exposure among radiation workers [12]. To our knowledge, no study has examined the sources of sex differences in the healthy worker survivor effect by analyzing component associations. Moreover, potential differences in the component associations using incidence data, rather than mortality or prevalence data, remain underexplored.

This study aims to explore the origins of sex differences in the healthy worker survivor bias by evaluating component associations using both mortality and cancer incidence data in a cohort of medical radiation workers. Identifying the source of potential sex differences in the healthy worker survivor bias will provide deeper insights into the mechanisms underlying the bias in occupational cohort studies.

## 2 Methods

The study population and methods have been described previously [12]. Briefly, the study population comprised all diagnostic medical radiation workers enrolled in the National Dose Registry (NDR) between January 1, 1996 and December 31, 2011 (*n* = 94,379). Workers with cancer or who had died before enrollment (*n* = 461) were excluded, resulting in a final analysis of 93,918 workers. This registry‐based linkage cohort study, which involved no direct contact with participants, was reviewed and approved by the Institutional Review Board of Korea University (KUIRB‐2019‐0092‐08).

Occupational exposure data were obtained from the NDR database, maintained by the Korea Disease Control and Prevention Agency since 1996 (https://www.kdca.go.kr/). Cumulative individual radiation badge doses (measured in Sievert) were calculated from quarterly badge readings recorded in the NDR based on Hp (10) measurements [13]. Occupational radiation exposure was categorized into no exposure (≤ 1 mSv) and exposure (> 1 mSv) groups based on the time‐dependent cumulative effective dose of up to each year. A 10‐year lag was applied a priori, considering the latency period between radiation exposure and chronic health outcomes [14]. Employment status was determined based on continuous badge records from the first and the last measurement date in the NDR.

Mortality data were obtained through linkage with Statistics Korea (http://kostat.go.kr/) using personal identification numbers. Deaths from all causes (ICD‐10 codes: A00‐Y89) and cancer deaths (ICD‐10 codes: C00–C99) were identified based on the underlying cause of death in the International Classification of Diseases, 10th Revision (ICD‐10). Cancer incidence data were obtained from the Korean National Cancer Center (http://www.ncc.re.kr/) through linkage to the centralized national cancer registry. Cancer cases were defined as the first primary malignant tumors (ICD‐10: C00–C99). Person‐years at risk were calculated from 1996 or the start year of employment (whichever occurred later) until the earliest of death, cancer diagnosis, or the end of follow‐up (December 31, 2018, for cancer; December 31, 2019, for mortality).

Associations for C1 and C3 were assessed using a stratified Cox regression model and a Cox proportional hazards model for counting process, respectively, to estimate hazard ratios (HRs) with 95% confidence intervals (CIs). The stratified model was applied for C1 due to violation of the proportional hazards assumption. In the C1 model, the exposure variable was defined as the dichotomized prior cumulative radiation exposure, and the outcome was survival time until leaving employment in the subsequent calendar year after the year of exposure. In the C3 model, the exposure variable was a time‐dependent employment status indicator, and the outcome was survival time until all‐cause mortality or cancer incidence. For the C2 model, logistic regression with generalized estimating equations was used to estimate odds ratios (ORs), with an independent working covariance matrix. Here, the exposure variable was employment status, with employed workers as the reference group, and the outcome was the dichotomized cumulative radiation exposure. Covariates included sex, attained age (continuous), birth year (< 1960, 1960–1969, 1970–1979, ≥ 1980), and employment duration (< 1, 1–4, 5–9, ≥ 10 years). Model adjustments were determined using deviance and the Akaike Information Criterion, consistent with previous analyses of the healthy worker effect [12]. An alternative binary exposure definition (i.e., 2 mSv) was applied to assess the robustness of the main findings. All statistical analyses were conducted using the *survival* and *geepack* packages in R software (version 4.3.3; R Foundation for Statistical Computing, Vienna, Austria).

## 3 Results

The association between prior occupational radiation exposure and time to leaving active employment (Component 1) was significantly elevated for all‐cause mortality (HR 1.06, 95% CI 1.02‐1.10) and all cancer incidence (HR 1.13, 95% CI 1.09–1.18) in male workers (Table 1). Conversely, prior radiation exposure was inversely associated with employment cessation in female workers (HR 0.82, 95% CI 0.78–0.87 for all‐cause mortality; HR 0.89, 95% CI 0.84‐0.94 for cancer incidence). In both male and female workers, adjusted ORs for the association between employment status and radiation exposure (Component 2) were significantly elevated for all‐cause mortality (OR 5.28, 95% CI 4.95‐5.64 for males, OR 2.41, 95% CI 2.25–2.58 for females). Similarly, HRs for the association between time‐varying employment status and survival time (Component 3) were elevated for all‐cause mortality in both male (HR 1.90, 95% CI 1.64–2.20) and female workers (HR 1.74, 95% CI 1.04–2.89). However, employment status exhibited a weak association with survival time for cancer incidence in both males (HR 0.95, 95% CI 0.83–1.08) and female workers (HR 0.89, 95% CI 0.73–1.08). This pattern persisted when alternative exposure definition was applied (Supporting Information).

**Table 1:** Assessment of the three component associations of the healthy worker survivor bias among medical radiation workers in South Korea.

Component 1 (ICD‐10)^a^	Total			Male			Female		
	No. of workers by employment		HR (95% CI)	No. of workers by employment		HR (95% CI)	No. of workers by employment		HR (95% CI)
	At work	Left work		At work	Left work		At work	Left work	
All causes of death (A00‐Y89)	23,843	7031	0.95 (0.92–0.98)	18,114	5271	1.06 (1.02–1.10)	5729	1760	0.82 (0.78–0.87)
All malignant neoplasms death (C00‐C97)	23,660	6773	0.94 (0.91–0.97)	17,942	5026	1.03 (0.99–1.07)	5718	1747	0.82 (0.78–0.87)
All malignant neoplasms incidence (C00‐C97)	21,419	6745	1.03 (0.99–1.06)	16,552	5079	1.13 (1.09–1.18)	4867	1666	0.89 (0.84–0.94)

Component 2 (ICD‐10)^b^	Total			Male			Female		
	No. of workers by cumulative dose		OR (95% CI)	No. of workers by cumulative dose		OR (95% CI)	No. of workers by cumulative dose		OR (95% CI)
	≤ 1 mSv	> 1 mSv		≤ 1 mSv	> 1 mSv		≤ 1 mSv	> 1 mSv	
All causes of death (A00‐Y89)	18,579	14,239	3.72 (3.55–3.89)	6927	8649	5.28 (4.95–5.64)	11,652	5590	2.41 (2.25–2.58)
All malignant neoplasms death (C00‐C97)	18,478	13,922	3.72 (3.55–3.89)	6842	8355	5.25 (4.92–5.60)	11,636	5567	2.41 (2.25–2.58)
All malignant neoplasms incidence (C00‐C97)	16,572	13,555	4.12 (3.91–4.33)	6198	8291	5.88 (5.48–6.32)	10,374	5264	2.63 (2.44–2.84)

Component 3 (ICD‐10)^c^	Total			Male		Female			
	No. of workers by survival status		HR (95% CI)	No. of workers by survival status		HR (95% CI)	No. of workers by survival status		HR (95% CI)
	Yes	No		Yes	No		Yes	No	
All causes of death (A00‐Y89)	32,126	692	1.90 (1.65–2.19)	14,965	611	1.90 (1.64–2.20)	17,161	81	1.74 (1.04–2.89)
All malignant neoplasms death (C00‐C97)	32,126	274	1.70 (1.37–2.10)	14,965	232	1.70 (1.35–2.13)	17,161	42	1.26 (0.59–2.70)
All malignant neoplasms incidence (C00‐C97)	29,178	949	0.96 (0.86–1.07)	13,946	543	0.95 (0.83–1.08)	15,232	406	0.89 (0.73–1.08)

Abbreviations: CI, confidence interval; HR, hazard ratio; ICD‐10, International Classification of Diseases and Related Health Problems, 10th Revision; N, number; OR, odds ratio.

^a^ HR of component 1 represents the hazard of leaving active employment among workers with prior cumulative exposure exceeding 1 mSv, compared to those with a cumulative dose of ≤ 1 mSv (reference group), assuming a 10‐year latency period.

^b^ OR of component 2 represents the likelihood of subsequent cumulative exposure status among workers who have left employment, compared to those currently at work (reference group), assuming a 10‐year latency period.

^c^ HR of component 3 represents the hazard of mortality or cancer incidence among workers who have terminated employment, compared to those who are still at work (reference group).

All models are adjusted for attained age (time‐varying, continuous), sex, birth year (< 1960, 1960–1964, 1965–1969, 1970–1979, ≥ 1980), and years of employment duration (< 1, 1–4, 5–9, ≥ 10).

## 4 Discussion

Our findings indicate that sex differences in the association between prior exposure and employment status are the primary contributor to the observed sex differences in the healthy worker survivor bias. In contrast, the direction of other component associations was consistent between male and female workers, suggesting that their contribution to sex differences was limited. Component analysis has been previously applied to assess evidence of the healthy worker survivor bias. This study extends its application by demonstrating its value in identifying the origins of the healthy worker survivor bias. To our knowledge, this is the first study to apply component analysis to determine the source of sex differences in the healthy worker survivor bias among radiation workers. Additionally, this study is strengthened by its linkage to comprehensive national mortality and cancer incidence registry data and the inclusion of all monitored South Korean male and female medical radiation workers. Given the substantial differences observed between male and female workers in component‐specific associations, sex‐stratified analyses may be essential to ensure accurate estimation of exposure‐disease associations in occupational cohort studies.

The differing patterns of association between prior radiation exposure and employment status (C1) in male and female workers can be explained by sex‐specific social and occupational factors. Among male workers, our finding of an increased risk of leaving employment following prior occupational exposure is consistent with most previous studies [1, 2, 3, 5, 6]. In contrast, female workers may leave employment for reasons unrelated to occupational exposure, such as marital status, household income, and childcare responsibilities [15, 16], which could weaken the association between prior exposure and employment status. Additionally, female workers may have left employment or been reassigned to lower‐exposure jobs due to a higher risk perception of radiation exposure compared to men in South Korea [17].

The significant association between employment status and subsequent exposure (C2), even after applying a 10‐year lagged exposure variable, supports the presence of the healthy worker survivor bias in both male and female workers. The difference in the magnitude of associations between male and female workers may contribute to sex‐specific differences in the degree of the healthy worker survivor bias observed in our cohort. However, since the direction of the association was consistent across sexes, its role in explaining the reported opposite patterns of sex differences in the healthy worker survivor bias [12] is likely limited.

The significant association between current employment and mortality (C3) observed in both male and female workers aligns with previous reviews suggesting that poor health is a major driver of early retirement [18, 19]. However, this pattern was less pronounced for cancer incidence, which is consistent with our prior findings indicating a stronger healthy worker survivor effect for mortality than for cancer incidence [12]. These findings demonstrate that the association between health status and leaving employment contributes to the healthy worker survivor bias, but this association had little influence on sex differences, as similar patterns were observed in both sexes. Since not all workers with poor health leave employment early, however, further research is warranted to examine the differentiated roles of health in the transition from work to retirement [20].

## 5 Conclusion

In conclusion, our findings indicate that sex‐specific differences in the healthy worker survivor bias stem primarily from the association between prior exposure and employment status among radiation workers. This study provides evidence for the origins of sex differences in the healthy worker survivor bias, utilizing both mortality and cancer incidence data. To better understand the sources of sex differences in the healthy worker survivor bias, future studies should incorporate sex‐specific component associations in occupational studies.

## Author Contributions

Won Jin Lee conceptualized the research and wrote the first draft of the manuscript. Jaeho Jeong performed the data analyses. Won Jin Lee and Young Min Kim supervised the data analyses and critically revised the manuscript. All authors contributed to the draft revision and approved the final manuscript.

## Disclosure by AJIM Editor of Record

John Meyer declares that he has no conflict of interest in the review and publication decision regarding this article.

## Disclosure

The authors have nothing to report.

## Ethics Statement

This is a registry‐based linkage cohort data, with no direct contact with participants. This study was reviewed and approved by the Institutional Review Board of Korea University (KUIRB‐2019‐0092‐08).

## Conflicts of Interest

The authors declare no conflicts of interest.

## Supporting information

SuppMat.

## Data Availability

The authors have nothing to report.
