# Supplementary material for: Component Associations of the Healthy Worker Survivor Bias in Medical Radiation Workers
Source: Am J Ind Med. 2025 Apr 25;68(6):552–6. doi: 10.1002/ajim.23727 (PMC12070152; doi:10.1002/ajim.23727)
Supplement: Supplementary file 1 — SuppMat. [file AJIM-68-552-s001.docx]

**Supplementary material**. Assessment of the three component associations of the healthy worker survivor bias among medical radiation workers in South Korea applying 2 mSv as alternative exposure definition

| Component 1 (ICD-10)^a^ | Total | | | Male | | | Female | | |
| --- | --- | --- | --- | --- | --- | --- | --- | --- | --- |
|  | N of workers by employment | | HR (95% CI) | N of workers by employment | | HR (95% CI) | N of workers by employment | | HR (95% CI) |
|  | At work | Left work |  | At work | Left work |  | At work | Left work |  |
| All causes of death (A00-Y89) | 19,114 | 5,295 | 0.98 (0.95-1.02) | 14,978 | 4,149 | 1.06 (1.02-1.11) | 4,136 | 1,146 | 0.85 (0.79-0.90) |
| All malignant neoplasms death (C00-C97) | 18,952 | 5,065 | 0.97 (0.93-1.00) | 14,826 | 3,927 | 1.03 (0.99-1.08) | 4,126 | 1,138 | 0.85 (0.79-0.90) |
| All malignant neoplasms incidence (C00-C97) | 17,473 | 5,102 | 1.07 (1.03-1.11) | 13,912 | 4,002 | 1.14 (1.09-1.20) | 3,561 | 1,100 | 0.94 (0.88-1.01) |
| Component 2 (ICD-10)^b^ | Total | | | Male | | | Female | | |
|  | N of workers by cumulative dose | | OR (95% CI) | N of workers by cumulative dose | | OR (95% CI) | N of workers by cumulative dose | | OR (95% CI) |
|  | ≤ 2mSv | > 2mSv |  | ≤ 2mSv | > 2mSv |  | ≤ 2mSv | > 2mSv |  |
| All causes of death (A00-Y89) | 21,995 | 10,823 | 3.13 (2.99-3.27) | 8,409 | 7,167 | 4.30 (4.05-4.57) | 13,586 | 3,656 | 1.93 (1.79-2.07) |
| All malignant neoplasms death (C00-C97) | 21,856 | 10,544 | 3.11 (2.97-3.26) | 8,295 | 6,902 | 4.28 (4.03-4.54) | 13,561 | 3,642 | 1.92 (1.79-2.07) |
| All malignant neoplasms incidence (C00-C97) | 19,786 | 10,341 | 3.26 (3.11-3.43) | 7,600 | 6,889 | 4.48 (4.20-4.77) | 12,186 | 3,452 | 2.01 (1.86-2.18) |
| Component 3 (ICD-10)^c^ | Total | | | Male | | Female | | |  |
|  | N of workers by survival status | | HR (95% CI) | N of workers by survival status | | HR (95% CI) | N of workers by survival status | | HR (95% CI) |
|  | Yes | No |  | Yes | No |  | Yes | No |  |
| All causes of death (A00-Y89) | 32,126 | 692 | 1.90 (1.65-2.19) | 14,965 | 611 | 1.90 (1.64-2.20) | 17,161 | 81 | 1.74 (1.04-2.89) |
| All malignant neoplasms death (C00-C97) | 32,126 | 274 | 1.70 (1.37-2.10) | 14,965 | 232 | 1.70 (1.35-2.13) | 17,161 | 42 | 1.26 (0.59-2.70) |
| All malignant neoplasms incidence (C00-C97) | 29,178 | 949 | 0.96 (0.86-1.07) | 13,946 | 543 | 0.95 (0.83-1.08) | 15,232 | 406 | 0.89 (0.73-1.08) |

N, number; HR, hazard ratio; OR, odds ratio; CI, confidence interval; ICD-10, International Classification of Diseases and Related Health Problems, 10th Revision.

^a^HR of component 1 represents the hazard of leaving active employment among workers with prior cumulative exposure exceeding 1 mSv, compared to those with a cumulative dose of ≤ 1 mSv (reference group), assuming a 10-year latency period.

^b^OR of component 2 represents the likelihood of subsequent cumulative exposure status among workers who have left employment, compared to those currently at work (reference group), assuming a 10-year latency period.

^c^HR of component 3 represents the hazard of mortality or cancer incidence among workers who have terminated employment, compared to those who are still at work (reference group).

All models are adjusted for attained age (time-varying, continuous), sex, birth year (<1960, 1960-1964, 1965-1969, 1970-1979, ≥1980), and years of employment duration (<1, 1-4, 5-9, ≥10).
